# A European Competence Framework for Industrial Pharmacy Practice in Biotechnology

**DOI:** 10.3390/pharmacy3030101

**Published:** 2015-07-29

**Authors:** Jeffrey Atkinson, Pat Crowley, Kristien De Paepe, Brian Gennery, Andries Koster, Luigi Martini, Vivien Moffat, Jane Nicholson, Gunther Pauwels, Giuseppe Ronsisvalle, Vitor Sousa, Chris van Schravendijk, Keith Wilson

**Affiliations:** 1 Pharmacolor Consultants Nancy (pcn-consultants), 12 rue de Versigny, 54600 Villers, France; 2 Callum Consultancy, 571 Berwyn Baptist Road, Devon, PA 19333, USA; E-Mail: ktcrowley@msn.com; 3 Vrije Universiteit Brussel (VUB), Laarbeeklaan 103, 1090 Brussels, Belgium; E-Mail: kdepaepe@vub.ac.be; 4 Department of Pharmacy, Waterloo Campus, King’s College London, 150 Stamford Street SE1, 9NH, London, UK; E-Mails: brian.gennery@kcl.ac.uk (B.G.); luigi.martini@kcl.ac.uk (L.M.); 5 European Association of Faculties of Pharmacy (EAFP), Department Pharmaceutical Sciences, Utrecht University, PO Box 80082, 3508 TB Utrecht, The Netherlands; E-Mail: A.S.Koster@uu.nl; 6 European Federation of Pharmaceutical Industries and Associations (EFPIA), Rue du Trône, 108, 1050 Brussels, Belgium; E-Mail: vivien.moffat@amgen.com; 7 European Industrial Pharmacists’ Group (EIPG), 4 avenue Ruysdael, Paris, France; E-Mail: jane@nicholj.plus.com; 8 Genzyme Flanders, Cipalstraat 8, 2440 Geel, Belgium; E-Mail: gunther.pauwels@Genzyme.com; 9 Pharmacy Department, Universitá di Catania, Viale Andrea Doria 6, 95125, Catania, Italy; E-Mail: giuseppe.ronsisvalle@unict.it; 10 Areta International, via Roberto LePetit, 21040 Gerenzano, Italy; E-Mail: vsousa@aretaint.com; 11 Vrije Universiteit Brussel (VUB), Laarbeeklaan 103, 1090 Brussels, Belgium; E-Mail: chrisvs@vub.ac.be; 12 Life and Health Sciences, Aston University, Aston Triangle, B75 6D9 Birmingham, UK; E-Mail: K.a.wilson@aston.ac.uk

**Keywords:** education, pharmacy, industry, Europe, biotechnology

## Abstract

The PHAR-IN (“*Competences for industrial pharmacy practice in biotechnology*”) looked at whether there is a difference in how industrial employees and academics rank competences for practice in the biotechnological industry. A small expert panel consisting of the authors of this paper produced a biotechnology competence framework by drawing up an initial list of competences then ranking them in importance using a three-stage Delphi process. The framework was next evaluated and validated by a large expert panel of academics (*n =* 37) and industrial employees (*n* = 154). Results show that priorities for industrial employees and academics were similar. The competences for biotechnology practice that received the highest scores were mainly in:

- “Research and Development”,
- ‘“Upstream” and “Downstream” Processing’,
- “Product development and formulation”,
- “Aseptic processing”,
- “Analytical methodology”,
- “Product stability”, and
- “Regulation”.

The main area of disagreement was in the category “Ethics and drug safety” where academics ranked competences higher than did industrial employees.

## 1. Introduction

The PHAR-IN (“*Competences for industrial pharmacy practice in biotechnology*”) [1] consortium consists of professional organisations representing industrial employees, *viz*, the European Industrial Pharmacists’ Group (EIPG) [2], and the European Federation of Pharmaceutical Industries and Associations, (EFPIA) [3], together with pharmacy academics from the European Association of Faculties of Pharmacy (EAFP) [4]. Amongst other projects, the PHAR-IN consortium looked at whether there is a difference in how industrial employees and academics rank competences for practice in the biotechnological industry. A small expert panel consisting of the authors of this paper produced a biotechnology competence framework by drawing up an initial list of competences then ranking them in importance using a three-stage Delphi process. The framework was next evaluated and validated by a large expert panel of academics drawn from EAFP (*n* = 37), and by industrial employees that were members of EIPG, EFPIA and European Federation for Pharmaceutical Sciences (EUFEPS [5]) (*n* = 154). 

The PHARMINE study (*Pharmacy Education in Europe*) found that a substantial number (37,308) of European pharmacists (6% of the total workforce) work in industry [6], this is similar to the world-wide figure of 10% given by the International Pharmaceutical Federation [7]. Recent publications have outlined the many profound changes in the drug industry, one of the most notable of which is a switch towards biotechnology [8,9]. The question arises, therefore, as to how to adapt pharmacy education to this switch towards biotechnology; this is under discussion in Europe [10], Australia [11] and the USA [12]. The PHARMINE study cited above found that in addition to the traditional courses in pharmaceutical technology (representing an average of 12% of contact hours of the five-year European pharmacy course), some pharmacy departments (10/31 studied) do give elective pre-graduate courses in industrial pharmacy but these are mainly concerned with chemical production of drugs and other matters such as regulatory affairs. The PHARMINE study also found that 11/31 departments give post-graduate courses in industrial pharmacy.

A potential mismatch may exist between the competences of recently graduated pharmacists and the professional requirements in industry if sufficient input from industrial stake-holders is not obtained. Furthermore, although many academics are actively involved in research, fewer are involved in the drug industry. The above situation requires that the educational system be capable of offering the right courses to produce the right person with the right competences at the right time. This paper presents a European competence framework for biotechnological practice in industry, the methodology by which this framework was produced, and the rankings of competences by industrial employees and academics.

## 2. Methodology

The competence framework was produced by the Delphi technique [13]. Delphi methodology has been used to produce consensus competence frameworks for healthcare professionals such as nurses [14] and medical doctors [15]; it has also been used to produce competence frameworks for secondary level teachers in biotechnology [16,17]. To our knowledge, Delphi has not been used to produce a competence framework for professionals working in biotechnology.

A small expert panel consisting of the 13 authors of this paper (six with an academic background, seven with an industrial background) produced a proposal for a competence framework, starting with an initial framework produced by two industrial consultants (BG and PC) and based on their expert knowledge and on recent literature in biotechnology. This version was then subjected to three Delphi rounds within the small expert panel producing a version that contained 46 proposals for competences in 13 categories. This competence framework was then evaluated and validated by a large expert panel consisting of (1) academics (members of EAFP), and (2) industrial employees (members of EIPG, EFPIA, EUFEPS and the Innovative Medicines Initiative (IMI) [18]) (using *surveymonkey* [19], see Appendix).

The large expert panel was invited to rank the 46 proposed competences using a uni-dimensional Likert ranking method [20] with a scale of 1 to 4. This scale with an even number of choices contrasts with scales with an odd number of choices that allow for a “neutral” option. The expert panel also had the possibility to check a “I am unable to rank this premise” box There was also the possibility of skipping a competence by not replying at all (blank).

The panel could also comment on the various competences.

## 3. Statistical Analysis

Response rates were calculated as the sum of the responses in ranks 1 through 4 divided by the total number of responses possible (=46 competences × 153 industrial respondents or × 35 academic respondents). Blanks and “I am unable to rank this premise” were pooled.

Ranking scores are expressed as means; this is for descriptive purposes only. Statistical significance was based on the Wilcoxon signed rank test for differences from the global median of the population (industrial employees or academics). The overall median was calculated from all answers given in order to make it possible to identify answers which score higher (or lower) than this overall median. Chi-square was used to compare results of academics with those of industrial employees.

Ordinal consensus was calculated using the Leik technique [21].

The statistical tests used are described on the GraphPad website [22].

## 4. Results

The distribution of academic and industrial respondents over the different European countries was not equal (chi-square = 72, d.f. = 1 and 19, *p* < 0.0001) (Table 1, top). There was no significant difference for age distribution; the main age groups represented were in the 41–60 years range (Table 2, bottom).

Table 1.Survey population characteristics.

**Table:** Country of residence.

	Industrial Employees		Academics	
	Number	%	Number	%
Country of residence				
Austria	1	0.6		
Belgium	12	7.8	5	13.5
Bulgaria	2	1.3	4	10.8
Czech Republic	1	0.6		
Denmark	5	3.2		
Finland	18	11.7		
France	15	9.7	1	2.7
Germany	8	5.2		
Greece		0.0	2	5.4
Hungary	1	0.6		
Ireland	8	5.2		
Italy	6	3.9	17	45.9
Malta			1	2.7
Portugal	14	9.1	1	2.7
Serbia	1	0.6		
Spain	1	0.6		
Sweden	3	1.9		
Switzerland	20	13.0		
The Netherlands	15	9.7	1	2.7
UK	23	14.9	5	13.5
Total	154 (1 did not reply)	100	37 (2 did not reply)	100

Chi-square = 72, d.f. 19, *p* value < 0.0001.

**Table:** Age group.

	Industrial Employees		Academics	
	Number	%	Number	%
Age group (years)				
18–30	13	8.4	3	7.7
31–40	29	18.7	9	23.1
41–50	46	29.7	13	33.3
51–60	54	34.8	9	23.1
61–70	11	7.1	4	10.3
>70	2	1.3	1	2.6
Total	155	100	39	100

Chi-square = 3.9, d.f. 5, *p* value 0.5617.

Overall ranking profiles (ranks 1 through 4) and response rates were not different between industrial employees and academics (Table 2).

**Table 2:** Frequencies of rankings (as % total possible) by industrial employees and academics of 46 competences for biotechnological professionals.

Rank	Industrial Employees (*n* = 153)	Academics (*n* = 35)
1	5.0	3.0
2	15.0	13.0
3	22.3	32.5
4	27.5	32.6
Blanks + “I am unable to rank this premise”	30.2	18.9
Total	100	100

Chi-squares on differences between industrial employees and academics regarding frequencies of ranks 1–4 = 2.12, d.f. 6, *p* value 0.9085.

Leik ordinal consensus based on the frequencies of ranking given in Table 2 was for industrial employees 0.50 and for academics 0.58.

Both academics and industrial employees ranked scores significantly above the global median for categories “Research and Development”, ‘“Upstream” and “Downstream” Processing’, “Product development and formulation”, “Aseptic processing”, “Analytical methodology”, “Product stability”, and “Regulation”. (Appendix Table A1). Only for competences “Describe the range of products available with recombinant DNA technology”, “Employ pharmaco-epidemiology skills, including the statistical methodologies to strategically evaluate a drug product and produce a risk management plan”, “Interpret clinical trial designs that address specific ethical issues e.g., in special patient populations” and “Design a consent process that ensures that subjects are not coerced into participating in clinical trials” was there a significant difference (chi-square) between academics and industrial employees—academics scoring higher than industrial employees. 

There were 59 comments on the following:

- The clarity of the survey
- The context within which answers should be given
- The specificity to biotechnology and not to industrial pharmacy practice in general
- The educational level (foundation or specialist) at which the competence would be acquired
- The balance between the relative importance of different competences

## 5. Discussion

### 5.1. Delphi Methodology and Statistics

The Delphi methodology implies that surveying be anonymous and so individuals were not targeted. This contrasts to the survey of pre-selected experts in which the answers obtained depends on the prior selection of the experts. Here, no limitations were fixed on the possibility to participate. Anonymity is a minor issue in this study as the Delphi method requires complete anonymity. In the PHAR-IN study, the identities of the participants were known to the authors but not to each other.

The Delphi technique was also double in that there was first a small expert panel three-round Delphi process and this was followed by a larger panel evaluation and validation. This contrasts to many studies that use the first step (e.g., Stupans, *et al.*,) without the second. It is similar to the methodology used by the MEDINE consortium. It has the advantage that Delphi results are validated by a large group of the professionals actually involved in practice (doctors in MEDINE, biotechnology employees in this PHAR-IN study) and by academics teaching biotechnology.

Regarding statistical analysis, there is an ongoing discussion on the use of parametric or non-parametric statistics when dealing with ordinal data such as Likert scales. Some studies use means and standard variations [23] others use medians (MEDINE). This paper proposes the use of means for descriptive statistics and medians (Wilcoxon) or frequencies (Chi-square) for analysis.

A final issue concerns consensus, which is at the centre of the Delphi process [24]. Leik ordinal consensus values were 0.50 (industrial employees) and 0.58 (academics). Using the scale given by MEDINE [25] this would be qualified as “moderate” consensus.

### 5.2. Profiles of Respondents

Response rates were high—around 70% for both categories—implying that those participating (both industrial employees and academics) were experienced and knowledgeable enough to reply. There was a significant difference in the distribution of participants across European countries with academics stemming mainly from Bulgaria and Italy and industrial employees from Finland and Switzerland. This discrepancy does not appear to be linked to concentration of pharmacy departments or the pharmaceutical industry in a particular country. Its effect on results is unknown. The UK had approximately equal percentages of academics and of industrial employees. There was no significant difference in age distribution. The main age groups represented were in the 41–60 years range suggesting that replies came from active and experienced participants.

### 5.3. Ranking Profiles

Categories linked to practical aspects concerned with production: “Research and Development”, “Upstream” and “Downstream” Processing”, “Product development and formulation”, “Aseptic processing”, “Analytical methodology”, “Product stability”, and “Regulation” received high ranking.

Preclinical and clinical categories scored lower with only two out of 19 competences receiving a score higher than the global median. Comments on these areas suggested that some of those replying considered such aspects as “general” competences not specifically linked to biotechnology practice.

Competences in the category “Ethics and drug safety” also received low scores especially on the part of industrial employees. Comments in this area suggested that this matter was self-evident *i.e.*, modern-day pharmaceutical clinical research standards prevent subjects from being coerced into participating in trials.

Finally, in only four out of 46 competences was there a significant difference between rankings of academics and industrial employees. This was mainly in the category “Ethics and drug safety” in which three out of five competences were ranked higher by academics.

## 6. Conclusions

Several competence frameworks have been proposed, for example, in the UK [26] and at the level of the international pharmaceutical federation [27]. This present study is different in that competences were ranked and validated by a wide European panel—of both industrial practitioners and academics—and were not only the fruit of the expert knowledge of a few. The results show that academics and industrial employees have very similar ideas on the relative importance of different competences for practice in the biotechnological industry. The competences rated as being of highest importance are those concerned with formulation, manufacture and quality control. Thus, academics appear to be in tune with industrial requirements.

## 7. Perspectives

The consensus framework presented here can be used by academics to examine whether the highly needed competences identified are in fact covered in their existing curricula. If these subjects are not covered in the course, such an examination will open up reflection and discussion on how to cover them. These competences are those of both technicians and research scientists. They may be partially acquired through extra-university and experiential learning on-the-job (APEL) [28]; biotechnological companies have their role to play in APEL—as they do in university master and Ph.D. programmes.

If readers of this paper would like to participate in the PHAR-IN project, they are invited to visit the PHAR-IN webpage [1] 

## Appendix

The PHAR-IN survey.

**Table A1:** Mean rankings by industrial employees (Ind.) and academics (Acad.) of the 46 proposed competences, arranged into 13 categories, for practice in the biotechnological industry (*n* = number of competence).

Number	Competence	Ranking	
n	1. Research and development.	Ind.	Acad.
1	Take an active role in a multidisciplinary team to interpret the key elements of a drug development strategy and use this to design early phase clinical studies	**3.3**	**3.4**
2	Understand the statistical principles used in preclinical and clinical research	2.9	3.1
3	Be able to critically review published studies in preclinical (including safety pharmacology) and clinical research.	**3.2**	**3.4**
	2. Preclinical sciences.		
4	Have an understanding of the choice and predictive value of the non-clinical testing programme as part of the overall drug development plan for chemical and biological compounds.	3.1	3.2
5	Be able to describe the general principles of non-clinical safety testing.	3.0	2.9
6	Know how non-clinical tests are integrated into the overall drug development plan (including scheduling of toxicology tests with respect to clinical trials).	3.1	3.2
7	Be able to use animal pharmacokinetics and toxicokinetics to inform the clinical development process.	*2.6*	2.8
8	Describe the importance of the selection of the preclinical animal model in order to have a better and more predictive non-clinical phase.	*2.8*	3.2
	3. Biological and advanced therapy.		
9	Describe the breadth of advanced therapy medicinal products (ATMPs) that are available and in development, including the scientific principles for the classification in to the categories of gene therapy, somatic cell therapy, tissue engineering and combined ATMPs.	2.9	3.2
10	Describe the range of products available with recombinant DNA technology.	*2.8*	3.3
11	Discuss the different needs between the pre-clinical and clinical trial needs of natural proteins and modified proteins	*2.8*	3.0
12	Describe the range of monoclonal antibodies available, and those in development, and discuss the potential long term safety issues with monoclonal antibodies.	3.1	3.3
13	Describe the global need for new and improved vaccines and the barriers to their development.	3.0	3.2
14	Define what a therapeutic vaccine is and describe how a therapeutic vaccine could influence therapy in a common disease area.	3.0	**3.4**
15	Describe what is a polysaccharide product and the regulatory and development challenges involved.	2.9	2.7
	4. Clinical pharmacology.		
16	Take an active role in a multidisciplinary team to design clinical pharmacology studies	2.9	3.3
17	Recognise the particular ethical issues of using non patient volunteers in clinical studies	3.0	3.2
18	Understand and interpret clinical pharmacodynamic and pharmacokinetic data especially that related to safety issues	**3.2**	3.3
19	Discuss how data from a clinical pharmacology study can inform the future development of a medicine	3.3	3.3
	5. Clinical development.		
20	Use pre-clinical pharmacology and safety data to prepare a clinical trial plan	2.9	3.3
21	Write a protocol for a study including the choice of design, the end points, whether to use a placebo and the inclusion and exclusion criteria	2.9	3.3
22	Interpret the elements of GCP that apply to the design and execution of clinical trials	3.2	3.1
	6. “Upstream” and “Downstream” Processing.		
23	Understand ‘’upstream’’ aspects of biopharmaceutical process development such as cell line development and generation and characterization of Master Cell Banks and Working Cell Banks, cell culture and harvesting	3.2	3.1
24	Understand ‘’downstream’’ aspects of biopharmaceutical process development such as isolation and purification of proteins	**3.2**	3.2
25	Identify Critical Quality Attributes (CQAs), and Critical Process Parameters (CPPs) and define a meaningful set of in-process controls and specifications to ensure quality and consistency of final product.	**3.3**	3.2
26	Have good working knowledge of the principles of “Comparability” as applicable to biopharmaceutical manufacturing changes.	**3.3**	3.1
	7. Product development and formulation.	****	
27	Understand the importance of defined quality standards for product and process components used in biopharmaceutical formulation and manufacture, and the potential for interaction with biopharmaceutical macromolecules.	**3.3**	**3.5**
	8. Aseptic processing.	****	****
28	Understand microbiological principles as they apply to sterility assurance in biopharmaceutical manufacturing.	**3.3**	3.2
29	Understand unit operations in aseptic processing and design of facilities and utilities in sterile manufacturing suite.	3.2	3.1
30	Understand concepts of Good Manufacturing Practice (GMP) and Good Distribution Practice (GDP) as applicable to the aseptic production, control, storage and handling of biopharmaceuticals.	**3.4**	**3.3**
	9. Analytical methodology.	****	****
31	Understand the principles, instrumentation and application of analytical methods (especially bioassay) used to characterize biopharmaceutical raw materials, intermediates and finished products.	3.1	**3.4**
	10. Product stability.		****
32	Understand the potential impact of environmental factors (such as temperature, light, oxidation) on biopharmaceutical proteins and consequences for product quality, safety and efficacy.	**3.4**	**3.4**
	11. Regulation.	****	****
33	Understand the regulatory framework applicable to the development, manufacture, quality assurance and testing of biopharmaceutical products	**3.5**	3.3
34	Use research skills to find regulatory documents used for the preparation of a Clinical Trial Application.	*2.8*	2.9
35	Use knowledge of specific legislation for biopharmaceuticals to review preclinical and clinical parts of a Marketing Authorisation dossier.	2.8	3.0
36	Make decisions based on regulatory and commercial information about what text should be included in a Summary of Product Characteristics and Patient Information for a biopharmaceutical.	3.0	3.0
37	Know how National Agencies conduct GXP inspections and how to prepare for them.	3.0	3.0
38	Have an appreciation of post-licensing responsibilities for drug safety and how to construct a risk management plan.	3.0	3.0
39	Understand the life cycle management of biopharmaceuticals	**3.2**	3.2
40	Understand the current regulatory requirements for biosimilars	3.2	3.1
	12. Ethics and drug safety.		
41	Analyse and report adverse event data from clinical trials	2.9	3.2
42	Employ pharmaco-epidemiology skills, including the statistical methodologies to strategically evaluate a drug product and produce a risk management plan	*2.5*	3.0
43	Interpret clinical trial designs that address specific ethical issues e.g., in special patient populations	*2.6*	3.1
44	Design a consent process that ensures that subjects are not coerced into participating in clinical trials	*2.5*	3.1
45	Utilise their knowledge to ensure that patient safety and patient education are priorities when either an originator biological molecule or a biosimilar molecule is dispensed in practice	2.9	3.2
	13. Commercialisation.		
46	Understand the significance of biomarkers as an integral part of the development process and economic evaluation of biopharmaceuticals	3.0	3.2

**Bold:** median for competence greater than global median of 3 (*n* = 4915 responses for industrial employees, =1305 responses for academics) (*p* < 0.05, Wilcoxon signed rank test) *Italics*: median less than global median (*p* < 0.05, Wilcoxon signed rank test). Grey coloured boxes refer to those competences in which at least one of the 2 scores was greater than the global median.
